# Healthcare professionals discourses on men and masculinities in sexual healthcare: a focus group study

**DOI:** 10.1186/s12913-023-09508-2

**Published:** 2023-05-24

**Authors:** Tommy Persson, Jesper Löve, Ellinor Tengelin, Gunnel Hensing

**Affiliations:** 1 School of Public Health and Community Medicine, Institute of Medicine, Sahlgrenska Academy, University of Gothenburg, Box 453, Gothenburg, SE-405 30 Sweden; 2 Knowledge Center for Sexual Health, Region Västra Götaland, Gothenburg, Sweden; 3 Department of Health Sciences, Rehabilitation Science, Mid Sweden University, Sundsvall, Sweden

**Keywords:** Masculinity, Sexual health, Attitude of Health Personnel, Focus Groups, Critical discourse analysis

## Abstract

Studies have reported that men’s uptake of sexual health services is low, that these services make them feel vulnerable, and that they experience sexual healthcare (SHC) as stressful, heteronormative, potentially sexualised and “tailored for women”. They also suggest that healthcare professionals (HCPs) working in SHC view masculinity as problematic, and situated in private relationships. This study aimed to explore how HCPs construct the gendered social location in SHC, specifically in terms of masculinity and a perception that masculinity is situated in relationships. Critical Discourse Analysis was used to analyse transcripts from seven focus group interviews with 35 HCPs working with men’s sexual health in Sweden. The study found that gendered social locations were discursively constructed in four ways: (I) by problematising and opposing masculinity in society; (II) through discursive strategies where a professional discourse on men and masculinity is lacking; (III) by constructing SHC as a feminine arena where masculinity is a visible norm violation; (IV) by constructing men as reluctant patients and formulating a mission to change masculinity. The discourses of HCPs constructed the gendered social location of masculinity in society as incompatible with SHC, and saw masculinity in SHC as a violation of feminine norms. Men seeking SHC were constructed as reluctant patients, and HCPs were seen as agents of change with a mission to transform masculinity. The discourses of HCPs risk othering men in SHC, which could prevent care on equal terms. A shared professional discourse on masculinity could create a common foundation for a more consistent, knowledge-based approach to masculinity and men’s sexual health in SHC.

## Introduction

Gendered health inequalities can be reinforced and reproduced by gender norms in healthcare regarding access to healthcare, patient-provider interactions and bias in health research [1–3]. Gender norms negatively affect the provision and quality of healthcare for women, e.g. biased assessments of women’s symptoms, women receiving less and cheaper treatments, fewer referrals and less rehabilitation than men with similar healthcare needs [4–6]. Men’s health, healthcare practices and communication also seem to be affected by gender norms, e.g. patients and healthcare professionals (HCP) perceptions of masculinity relate to men’s health seeking and interactions between patients and HCPs [3, 7–9]. Gender norms are created and reinforced in a number of ways [10], such as through discourse, i.e. how gender and health are communicated in speech and writing [11–13]. Other ways include institutional policies, values, and attitudes (e.g., in families, schools, and workplaces), and individual opportunities, experiences, and behaviours [1, 10]. In healthcare, gender-based discourses seem to play a role in the quality of care provided [8, 14–16], and may create and reinforce gendered health inequalities and biases. Gender-based discourses can be understood as HCPs attitudes coming into play in direct interactions with patients e.g. more friendly towards patient of a certain gender or more eager to find solutions [17].

The practice of medicine and healthcare institutions have been described as male biased, taking men and masculinity as the norm, i.e. that men and masculinity are viewed as normal or neutral at the expense of women and femininity [18], but there are exceptions. Sexual healthcare (SHC) has traditionally been regarded as an issue for women [19, 20], but there is a growing awareness that men should be included in sexual health programmes [21–23] and services [24]. Such services should provide information, counselling, testing and treatment regarding sexually transmitted infections, unwanted pregnancies and sexualised violence. As well as information about sexual development and treatment and care for sexual dysfunction, e.g. erectile dysfunction and premature ejaculation [23, 25, 26]. Studies from the U.S., Britain and the Nordic countries have reported that men’s uptake of sexual health services is low [20, 27–32], that these services make them feel vulnerable [33], and that they experience SHC as stressful [34], heteronormative [35], potentially sexualised and “tailored for women” [36].

A Swedish population-based survey [37] identified that 42.1% of men who had sought SHC reported that it had only partially helped them, and 23.3% said it had not helped them at all. In the Nordic countries, including Sweden, men’s access to SHC is limited by organisational barriers e.g. lack of prioritizing men’s SHC, lack of holistic approach to care and HCPs experiencing unclear mandates for working with men’s SHC [32]. SHC for men in Sweden has been described as being in everyone’s interest but no one’s responsibility [38].

Men have been cited as missing clients in sexual health [39, 40]. Previous research has focused on how men avoid seeking SHC [41–44], which is often explained as men adhering to norms of masculinity [45–47]. Masculinity norms places expectations on men to be stoic, tolerate pain and illness and to avoid seeking professional help [48, 49]. Adhering to these norms have been associated with beliefs and behaviours that increase health risks [46, 50] including sexual risk-taking, such as the number of sexual partners and attitudes to condom use and to testing for sexually transmitted infections [51, 52].

Masculinity, according to Schippers [53], is a social location. This is not strictly a place, but a position that can be embodied by men, women and others through a combination of masculine practices and attributes in social interactions. The location is constructed in relation to femininity and un-masculinity in all social relations, including institutional practices at clinics. Social location can also be understood as a person’s position within a gender regime, i.e. how genders relate to each other within an institution [54]. This perspective assumes that there are different forms of masculinity and femininity [54]. In a previous study, we identified that HCPs perceived men and masculinity as a potentially challenging in SHC, i.e. that the social location of masculinity could be problematic for both patients and HCPs. We also found that HCPs’ notions of masculinity were situated in relationships, i.e. that HCPs understood masculinity in a context of their own intimate, private and sometimes sexualised relationships [55], whether these were real or hypothetical. The study showed how the gendered social location of masculinity was perceived in SHC. However, there is a lack of knowledge about how this social location is constructed, and how HCPs’ discourses on men and masculinity relate to how it is constructed. The conceptual framework in this article is inspired by critical discourse analysis (CDA), where discourses are viewed as communicated “systems of meaning” that can create change, and which construct and shape shared understanding and social realities, regardless of the speaker’s intent [56]. CDA examines how meaning about different phenomena, such as gender or masculinity, is constructed through language within a specific context and has been described as useful in “examining how dominant discourses construct health issues” [57].

This study aimed to explore how HCPs construct the gendered social location in SHC, specifically in terms of masculinity and masculinity seen as situated in relationships.

## Method

### Study design

This study was designed as an explorative, qualitative focus group study. Focus group discussions offer an understanding of group norms, facilitate individual disclosure and provide connections between local discourses, group norms and societal discourses [58–60]. The data were analysed using discourse analysis. We choose to focus on discourse as language, i.e. text and speaking, viewed as a “communicative event” and as part of a social process connected to other aspects of social life [61]. We used a model from CDA, a form of discourse analysis which focuses on how language can change and reproduce power, as it is suitable for revealing how acts of interpersonal communication produce and maintain power and knowledge, how they shape experiences, activities, relationships and identities in relation to the setting, and how they construct social practices and structures [61].

### Recruitment and study sample

The study sample consisted of HCPs working with men’s sexual health either at primary healthcare centres (PHCCs) or at clinics specialising in sexual and reproductive health. To obtain broad variation in experiences, HCPs from different types of clinics were invited to participate, as well as from a variety of occupational categories and from different clinic catchment areas. The inclusion criterion was that participants should be working clinically with men’s sexual health. To facilitate natural conversation and self-disclosure, we assumed that participants would be more comfortable in groups who worked together.

Sexual and reproductive health clinics were contacted using the inter-organisational strategic Sexual and Reproductive Health and Rights (SRHR) network in the Region Västra Götaland, Sweden. Clinics included sexual health clinics, STI clinics, antenatal clinics, clinics for gynaecology and venereology, youth clinics and PHCCs. Requests to participate in the study were sent through newsletters and letters to key stakeholders. Nine clinics expressed interest in taking part, but three chose not to participate due to time constraints. As none of the other six clinics was a PHCC, and as PHCCs are important providers of adult men’s SHC, it was deemed important to include at least one PHCC in the study. Thirty new PHCCs were contacted directly, and were suggested by Research and Development primary healthcare in the RegionVästra Götaland. None of them chose to participate. A new letter was sent to PHCCs through the SRHR network, resulting in one PHCC choosing to participate. In total, 35 HCPs were interviewed in seven focus groups conducted at seven different clinics. All participants were working with men’s sexual health. See Tables 1 and 2 for the characteristics of the participants.

**Table 1:** Characteristics of participants

Characteristic	n
No. of participants	35
Gender, n (%)	
Female	24 (68.6%)
Male	11 (31.4%)
Age range	29–71 years
Profession, n (%)	
Assistant physician	1 (2.86%)
Counsellor/social worker	5 (14.29%)
General practitioner	3 (8.57%)
Midwife	12 (34.29%)
Nurse	7 (20%)
Nursing assistant	3 (8.57%)
Psychologist	4 (11.43%)
Type of clinic, n	
Primary healthcare clinic	1
Venereological clinic	1
Youth clinic	3
Reproductive clinic	1
Men’s sexual health clinic	1
Catchment areas, n	
Inner-city	2
Suburbs	2
Smaller towns	2
Rural area	1

**Table 2:** Gender distribution by profession

Gender distribution by profession	Women, n	Men, n
Assistant physician	1	-
Counsellor/social worker	3	2
General practitioner	1	2
Midwife	12	-
Nurse	3	4
Nursing assistant	2	1
Psychologist	2	2

### Data collection

Data were collected through seven audio-recorded focus group interviews, consisting of four to six participants each. To ensure privacy, the focus groups took place in rooms at the participants’ place of work, and they lasted approximately 90 min. The focus groups were moderated by the first author and co-moderated by four different public-health researchers. The co-moderators were only involved in the data collection. Their task was to explore statements and claims made during the focus groups which might have been overlooked by the moderator. Notes were taken by both moderator and co-moderators during the focus groups. The moderator initiated the focus group interviews using a guide (Table 3), and participants were encouraged to discuss freely and share their thoughts, experiences, and views on the topics. Probes and follow-up questions included: *Do you all agree with this? Can you give an example?* and *What do the rest of you think about this*? These were used to encourage examples and clarification, and to facilitate and stimulate communication and discussion within the group.

**Table 3:** Focus group interview guide

Main questions
Tell us about the men who come here seeking sexual healthcare.
What is it like to meet men seeking sexual healthcare?
What is masculinity to you?
What is your perception of the men that come here?
Are there qualities that you consider to be masculine or un-masculine?

During data collection we did not provide any definition or examples of masculinity to the participants, instead participants were free to define and exemplify masculinity based or their own notions. The focus group interview guide was pilot-tested at two clinics. No changes were made to the questions, but probes and follow-up questions were adjusted to encourage inclusion and discussion. Data from the pilot focus groups were not included in the analysis or the results.

### Ethical considerations

The Regional Ethical Review Board in Gothenburg, Sweden (registration number 543 − 14) approved the study. All participants gave written, informed consent, and were informed verbally and in writing that they could withdraw from the study at any time. Names of, and detailed information about participants and clinics have been removed from the study.

### Data analysis

The data in this study were previous used in a study on HCPs’ notions about men and masculinity [55]. This study, taking a discourse perspective, offers a secondary analysis of the focus groups interviews. Data from the focus group interviews were transcribed verbatim by a professional transcribing firm. All transcripts were proofread and corrected for any errors by the first author (TP) to ensure quality and accuracy. Before coding, the transcripts were closely read and re-read to re-familiarise the authors with the text. The texts were then coded using Fairclough [61] three-dimensional model as inspiration. This model consists of three interrelated dimensions of discourse: (a) a text-level, (b) discursive practices and (c) social practices. Codes were identified as sections of text where HCPs described, through their discourse: (1) SHC as a gendered social location, (2) the social location of masculinity and (3) an understanding of masculinity as situated in relationships. The analysis was undertaken in three stages (see Table 4), starting with (a) identifying key words and phrases that showed how HCPs constructed attitudes, opinions and values. The second stage (b) analysed discursive practices in the text to identify how words were put together in sentences to create change. This included how topics were discussed and interpreted, what values and opinions the sender conveyed to the recipient, and how the speakers positioned themselves as subjects. This stage also analysed discourses involved in group dynamics and organisational cultures, and the use of interdiscursivity (how different discourses are incorporated into the same statement). The third stage (c) analysed social practices, i.e. an analysis at the norm level, including the wider societal, ideological, and political context in which the communication was taking place. Coding at this level identified how language created social relationships and practices. It included looking at language as a form of power, and communication as a social event where language and choice of words form the context of social community. Finally, codes from all three stages were aggregated in broader themes that showed how the linguistic microlevel, i.e. the words and sentences, interacted with norms, traditions and ideologies within the organisation and in society.

**Table 4:** Example of analysis

Example code	Dimension 1: Text	Dimension 2: Discourse practice	Dimension 3: Social norms
*“Obviously if I am talking with a man than perhaps that man has to explain a bit more to me than if I am talking with a woman. I perhaps need to allow the man to talk a bit more… and explain a bit more*, *descriptively*. *Because if it is a woman then perhaps, I think I know. “*	Keywords: The word “Obviously” shows that the speaker presents the statement as self-evident. The word “descriptively” is used to emphasize that the speaker’s preunderstanding of men’s and women’s sexual health issues differ.	The speaker compares differences in how they communicate with patients based on whether the patient is a man or a woman as self-evident. The speaker interdiscursively links the gendered differences in communications to differences in preunderstanding of patients’ sexual health issues based on patient’s gender.	The speaker constructs differences in interactions between male and female patients in SHC as self-evident illustrating a binary perception of gender. The statement relates to the discourse on the lack of training and education on men’s sexual health among HCPs who work with men’s SHC.
*“But I also think that it is our mission to make them* [men seeking SHC] *secure in different ways, sort of.”*	Keywords: The phrase “our mission” indicates that the speaker portrays the statement as a task that the clinic is charged with. The word “secure” is presented as an aim for men who seek SHC.	Men seeking SHC are implicitly described as being insecure. By using the phrase “our mission” the speaker indicates that making men secure is an important part of providing SHC for men, rather than a personal opinion.	Being, feeling and making others feel secure is used by participants in descriptions of masculinity in relationships, i.e., participants notions of desirable and preferable masculinity. By presenting men seeking SHC as insecure patients are portrayed as not performing desirable masculinity, and HCPs as having a mission to change patients’ masculinity.

To increase reliability [62] the data were initially coded independently by TP and ET. The codes were compared to identify broader recurring themes in the focus group interviews [63]. As a cisgender man working strategically with SRHR, TP was subject to preconceptions which were largely based on an organisational discourse where an aim was to report an increase in the proportion of men taking part in SHC. The co-analyst ET approached the data as a cisgender woman with a background in gender-related health-inequity issues, specifically issues around gender norms.

## Results

The gendered social location was constructed in four discursive ways (i.e. themes): (I) Problematising and opposing masculinity in society, (II) Discursive strategies compensating for the lack of a professional discourse on men and masculinity, (III) Constructing SHC as a feminine arena where masculinity is a visible norm violation, (IV) Constructing men as reluctant patients and formulating a mission to change masculinity.

### Theme I: problematising and opposing masculinity in society

SHC was constructed as separate and in opposition to norms of masculinity in society. Men seeking SHC were described as presenting “societal masculinity” at the clinic. Participants distanced themselves from masculinity in society by construing it as problematic, harmful and wrong for SHC and society as a whole. Apart from the oppositional discourse, HCPs did not share an alternative professional discourse regarding masculinity or men in SHC.

#### Distancing from societal masculinity

Participants positioned SHC discursively as being in opposition to, or distanced from masculinity in society, which was described as problematic. Factual modalities were used to construe SHC as “*actually being*” a sub-culture which was separate from society. Participants distanced themselves semantically from societal masculinity by using vocabulary such as “*traditional*”, “*stereotypical*”, “*that*”, “*normative*”, “*the classic*” or “*the classic image of*” masculinity. A key word in this process was the word “*macho*”. “*Macho*” was used both as a synonym for masculinity in society and to illustrate problematic aspects of masculinity in society. Macho and “*macho culture*” were linked to descriptions of men’s domination, and to degradation of women and transgendered people. The terms were associated with words and phrases such as “*rape culture*”, “*acting out*”, “*inability to communicate*”, “*inability to seek help*”, “*power*”, demonstrating “*prerogative of interpretation*”, and being “*disrespectful*”, “*condescending*” and “*tough*”. Participants’ critical attitudes towards masculinity in society were portrayed as different from, or better and truer than the attitudes of patients, other areas of healthcare and the media. A critical attitude was described as a prerequisite for working with men’s sexual health. Emotional modalities, such as “*I feel that*” or “*in my opinion*”, were used to describe masculinity in society as detrimental to the SHC context. In descriptions of men as patients, HCPs portrayed them as introducing, performing, or presenting societal masculinity at the clinic.

“*I think that most *[men who come seeking SHC] *who I have spoken with about what masculinity entails say that you* [as a man] *are expected to be aggressive, that you should assert your position and that you should be superior to women and so on.*” (FG 7, Men’s sexual health clinic).

This form of masculinity was said to be a “*caricature*” of masculinity, and harmful to patients. Patients were described as trying to “*live up to*” societal masculinity and failing. This failure was construed as an underlying reason for the fact that many patients needed SHC.

#### The lack of a professional discourse

Apart from an opposition to masculinity in society, there was no explicitly shared formalised discourse on masculinity or approach to men as patients. Participants were aware that they lacked a shared approach, and problematised their own oppositional discourse by describing it as intellectualised and not anchored in patients’ reality. This intellectualised approach to talking about masculinity was considered to be ineffective in clinical interactions. Patients’ masculinities were described as problematic and harmful, but simultaneously a better reflection of reality than the intellectualised discourses.

*“And it would be easier not to understand masculinity perhaps, but if we had a clearer pattern, men come and women come and we meet them roughly the same way, but if we had a clearer, how should we …, so that we felt comfortable meeting men, then it would be easier to understand. Well maybe not masculinity but that masculine aspect of seeking* [SHC] *from us.”* (FG 5, Reproductive clinic)

Not having a shared discourse on masculinity also meant that HCPs lacked a shared professional discourse for interaction with men seeking SHC. The absence of a professional discourse was explained as being the consequence of inadequacies in formal training and education, and a lack of clinical experience with men. Participants at clinics with a lower uptake of men and participants whose professional training focus more on women’s sexual and reproductive health expressed this lack more strongly. Participants used formal and emotional language to express the lack of a professional discourse. Examples of key phrases in the formal discourse included “*having received little*” and “*I am lacking that competence*”. Examples of emotional language included “*I feel insecure*” and “*I feel I am very much lacking*”. The lack of a professional discourse was particularly clear in descriptions of men outside HCPs’ notions of normative masculinity, such as trans men and non-heterosexual men.

### Theme II: discursive strategies to compensate for the lack of a professional discourse on men and masculinity

HCPs used discursive strategies to compensate for the lack of a professional discourse on men and masculinities in SHC. These strategies included connecting, recontextualising and re-negotiating existing discourses. Discourses which expressed private attitudes were presented as unprofessional but aligned with SHC’s opposition to masculinity in society. They were therefore considered acceptable for use as tools in a professional context. HCPs’ relationally situated understanding of masculinity, i.e. notions of masculinity based on HCPs’ own intimate, private and sometimes sexualised relationships, were validated by referring to clinical experiences of men. Masculinity and the importance of masculinity in SHC were minimised or denied in the sense that HCPs considered themselves gender-neutral in a professional capacity, which negated the need for a professional discourse on men and masculinity.

#### Legitimising private attitudes - unprofessional but oppositional

Personal or private attitudes, such as understanding masculinity as relationally situated, were explicitly and implicitly described as unprofessional and undesirable in SHC. This was evident in the fact that HCPs linked professionality with “*not mixing in*” private attitudes or emotions. Implicitly private attitudes were described as seeping into, and “*affecting*” professionality. Participants described how they attempted to avoid private discourses on masculinity. They distanced themselves from having private, “incorrect” attitudes by making use of silence, irony and self-distance. However, to oppose or disassociate themselves from societal masculinity, HCPs used discourses on masculinity which expressed a personal and relational understanding of it, or attitudes towards it. In this way, personal attitudes were aligned with SHCs’ opposition to societal masculinity, and were thereby legitimised as part of an oppositional strategy. At the same time, HCPs implicitly reproduced societal discourses on masculinity in personal, relationally situated discourses.

Participants’ discourse involved an “embodied understanding”, in which their own gender was constructed as a professional tool for understanding or relating to patients’ sexual health. To describe women’s sexual health, female HCPs used vocabulary such as “*easier to relate to*”, “*naturalness*” and “*we women*”, indicating a shared understanding of women’s sexual health and femininity which was lacking in their discourse on men’s sexual health and masculinity. This discourse reframed personal gendered attitudes as professional. Male HCPs did not explicitly describe an embodied understanding of male patients. Instead, they described receiving validation from male patients based on their gender expression, and said that male patients need identification based on sharing their gender with HCPs. Some male HCPs spoke of an embodied opposition to masculinity in society.

“*I identify as a man and* [I] *am therefore masculine, but do not want to be associated with the qualities that are opposed to women and femininity* […] *My notions of traditionally feminine qualities* [are that they] *involve qualities that are good, such as caring, an ability to reflect, being given to emotions, softness, and speaking of whether this* [discourse] *is on an intellectual plane or not. My very existence challenges the sort of masculinity that is based on that contrast*.” (FG 7, Men’s sexual health clinic)

#### Validating relationally situated masculinities through the experiences of men

Empirical descriptions of men as patients were used to negotiate whose opinion of masculinity and whose attitudes towards it were most valid. Both male and female HCPs used closeness to men, and experience of men as patients to defer or establish prerogative of interpretation. In these negotiations, participants used examples that reflected private and relationally situated perceptions of masculinity. “The patient” was thus used as a discursive legitimiser in terms of validating personal attitudes as professional observations. Similarly private closeness to men was used to validate attitudes about men and masculinity.

A^1^: *“Yes, no, I am thinking that I live in a house with five men: four sons and a husband.”*

B: *“You should know …”*.

A: *“So I should know* [what masculinity is].” (FG 5, Reproductive clinic).

In mixed gender focus groups, male HCPs referred to clinical experiences or observations of other men, even when other participants specifically asked about their opinions based on their gender.

#### Denying or minimising masculinity

A strategy used to compensate for the lack of a professional discourse and relationally situated discourses involved minimising or denying masculinity, or the importance of masculinity, in SHC. This manifested itself in a discourse where HCPs claimed to be gender-neutral in a professional capacity. Instead of referring to patients as ‘men’ or ‘masculine’, they referred to them in an SHC context as people/patients/humans/de-gendered/gender-neutral.

A: *“Does this mean that you don’t perceive whether a person is masculine or feminine?”*

B: *“Yeah but, we do actually. I actually do.”*

C: *“But I never think that way.”*

B: *“No, I don’t think that way, but of course when I look at you, I don’t think masculine, I don’t do that. […]”* (FG 1, Venereological clinic)

Men’s sexuality and patients’ masculinity were discursively reduced or “objectified” to physical aspects such as genitals, and were constructed as mechanical or technical. The idea of perceiving patients as gendered was described as unfamiliar in SHC. The gender-blindness discourse was described as a consequence of working in SHC. For example, this appeared in statements about how male patients they had encountered clinically were all so different, and had such a variety of problems, that it was impossible to talk about masculinity. There seemed to be a contradiction between idealised statements and experiential descriptions. HCPs suggested that the correct or ideal way of relating to patients was to perceive or assume gender-blindness while “*treating everyone the same*”, but they also said that they assumed and acted on gender differences in clinical interactions with patients.

### Theme III: constructing SHC as a feminine arena where masculinity is a visible norm violation

SHC was discursively constructed as a feminine arena by referring to the profession, as well as its history, tradition and practice, as feminine, i.e. an institution predominantly for, and consisting of women. The social location of masculinity in the arena was construed as a violation of norms. Discourses on the waiting room demonstrated how men and masculinity were made visible by not fitting in, and that SHC needed to be de-feminised to be made more accessible for men.

#### Constructing sexual health as a feminine arena

SHC was discursively constructed as a feminine arena. Femininity was portrayed as an ideal, or as something “good” in the context. Regardless of their gender, participants described having been “*educated*”, “*trained*”, “*indoctrinated*” or “*imprinted*” to regard SHC as feminine, and associated their clinical practices with key words like “*nurturing*”, “*mothering*” and “*women’s role*”.

*A: “*[…]* but I think it’s not just education that guides us in what we view as, that we see women and femininity as good.”*

*B: “Mm.”*

*A: “Yes, but surely, I absolutely have to say that I have been imprinted, and that I see that as a positive imprint *[as a consequence]* of the fact that I, as a nurse, have seen more examples of female role models than *[are seen]* in society at large. And I am glad about that.”* (FG 7, Men’s sexual health clinic)

SHC was considered to be traditionally and historically linked to women’s reproductive health, and regarded as a women’s issue. The SHC profession was constructed as female by making guesses about gender statistics, stating outright that the workforce was predominantly or only made up of women, or by referring to HCPs in SHC as “*other women*”. A typical HCP was described as a female midwife. Participants spoke of the importance of gender diversity, and the need for changes in recruitment strategies to allow other professions to work in SHC, implying a need to recruit more men into the field. Men working in SHC described how they were perceived by patients and colleagues as doing something outside the norms of masculinity, but they also described how they received overwhelming gender-based validation and had difficulties handling gendered expectations from men who were seeking SHC. It should be noted that some participants stated vehemently that they did not wish to associate gender with their professional role, and that the gender of HCPs should not matter.

*”But then that is actually the image I have* [of HCPs], *since we are actually mostly old ladies. So I guess that’s just the way it is.”* (FG 5, Reproductive clinic)

Organisational structures, such as the focus and availability of types of clinic and medical specialisation, earlier experiences, formal training and education, were all described in a way which assumed that SHC was feminine and aimed at women. An example of this involved statements about the need for routines and further training aimed at not putting men at a disadvantage.

*”And then, or sometimes, that is almost always, their* [men’s] *visits are about getting tested, but other things tend to come up, I feel. And I can feel that I find it difficult to give them answers because I don’t have that education, because I, yes in midwifery training you study women and women’s sexuality and women’s sex, but nothing about men. And here we have, I have, received a one-day training session* [on men’s sexual health], *so mostly I don’t feel I can meet their needs.”* (FG 2, Youth clinic)

#### Masculinity as a violation of feminine norms

Discourses on masculinity in SHC were juxtaposed with a discourse involving SHC as a feminine arena. The fact that HCPs identified themselves and the arena from a feminine perspective was used to construct the social location of masculinity in SHC. It contributed to masculinity being seen as not preferable, potentially problematic, provoking, uncomfortable, ignorant and inaccessible to female HCPs. SHC clinics specifically aimed at men were described as “separatist rooms”^2^ for men. Equally, masculinity was construed as a norm violation, particularly patriarchal notions of masculinity such as men’s power over women, and women were construed as the norm, implicitly and explicitly. Women were explicitly cited to describe normal clinical situations and communication, to illustrate what it was like to meet men. Examples of key phrases in the women-as-norm discourse included “*Of course we are specialised in women*”, referring to women as a “*frame of reference*” or “*generally speaking*”, and men in SHC as “*doing something un-masculine*”.

*A: “We have a frame of reference for how a pap-smear test should be. We have seen thousands of women, all of us *[have]*. We have a very large frame of reference, and then a man comes along and you are expected to … that …”*

*B: “Yes, you can’t tell what’s normal because you haven’t seen enough *[men]* … so, sure.”* (FG 5, Reproductive clinic)

Women seeking healthcare was described as “*taking responsibility*”, socially expected, naturally socialised, “*much easier*” and “*routine*”. Men were considered to seek SHC because they had failed in something, such as using a condom, or alternatively “*when it came up*” in relation to other health issues at PHCCs, such as urinary problems. Female HCPs expected men to describe sexual health issues more clearly, as they did not share the embodied experience of being a woman. When men sought SHC, it was described as more aggressive and threatening than when women sought it. Men’s presence was said to generate insecurity and challenge the professional role and competence of HCPs. Implicitly, it was almost impossible to speak about masculinity in SHC, particularly without comparing it to the experience of women. Men were described as “*taking up space*”. Interactions with men were described as metaphorical “*struggles*” or “*confrontations*” which could negatively affect the ability to treat patients. Women’s sexual health was described as “*natural*”, “*holy*” and comprehensible. Men’s sexual health was associated with less common problems. In comparing interactions with male and female patients, phrases such as “*I am better at*” were used in terms of interactions with female patients, and “*I am worse at*”, “*I would like to be better at*”, “*I help them less*” and “*I sometimes become a worse therapist*” were used in terms of interactions with men. Communication with men was considered to be “*something the whole clinic has to work at*”. This indicates that the professional discourse was based on women.

“*But it would be great if we had a, that is, a form of structure* [for providing SHC for men], *then you can always divert from that, but if you have nothing all you can do is divert.”* (FG 5, Reproductive clinic)

Masculinity as a norm violation was also expressed through a heterosexual discourse. Men’s presence at the clinic, and physical examinations of men, were described as potentially sexually “*charged*” and “*intimate*” interaction between HCPs (implicitly or explicitly identified as female) and men as patients. These interactions were described as unwanted, as well as problematic for men and in relation to men.

#### Making masculinity visible through discourses on the waiting room

The waiting room was a discursive arena where masculinity was made visible. Participants said that patients’ gender identity could be more important in the waiting room than in the examination room. Waiting rooms were described as being, or having been, very feminine. Participants described needing to change this situation by de-feminising waiting rooms, e.g. the colour of the walls, the artwork and campaign posters, to compensate for the fact that the clinic was dominated by women. They felt this would make it into a place men could “*feel*” was for them as much as for women. Collecting or calling out patients’ names in the waiting room was described as a gendered situation. Including the man’s name when heterosexual couples were called was considered to be an inclusion strategy. The woman, as primary patient, was called first, and then the man, to make him feel included. Participants described feeling uneasy and overanalysing when they called a trans man in a waiting room filled with women. The waiting room was also constructed as a potentially sexually charged place. Older female HCPs described how men visibly relaxed when they realised it was not a young woman coming to collect them. Participants discussed shared experiences of how they assessed different masculinities in the waiting room by analysing where and how the patients were sitting and what they were wearing. Men in the waiting room were portrayed as disruptive to other patients and HCPs. They were described as feeling “*shitty*” if they had to sit with other men, and “*courageous*” if they were the only man in a waiting room full of women.

”*I don’t think that everyone at the clinic finds it as amusing when five guys come in and act a bit macho and want condoms and asks what condom the receptionist thinks is best, sort of and similar things.”* (FG 2, Youth clinic)

### Theme IV: constructing men as reluctant patients and formulating a mission to change masculinity

HCPs’ discourses on men seeking SHC were linked interdiscursively to organisational expectations of a need to report an increase in the proportion of men seeking SHC. HCPs expressed mixed attitudes involving positive feelings towards men seeking SHC and giving them undue praise for coming in. These discourses constructed men as reluctant or missing patients. In descriptions of masculinity as an underlying reason for men seeking SHC, participants expressed an underlying mission to change masculinity, and presented HCPs as agents of change.

#### Referencing discourses on men as missing patients

A: *“Yes, precisely that*, [men] *probably feel that it is a bit unnecessary to communicate, but then still when they finally* [come to the clinic] …*”*

B: *“Exactly.”*

A: *“… and finally make it over the threshold or in some way end up with someone like someone here … most of them consider it a gift.”* (FG 6, Youth clinic)

Descriptions of men and masculinity in SHC were contextualised interdiscursively by using or referencing organisational discourses and institutional expectations of a need to report an increase in the proportion of men seeking SHC. Participants expressed ambivalence in their descriptions of men who seek SHC. Their vocabulary reflected positive emotional attitudes such as “*liking it*”, “*feeling stimulated*”, “*happy*”, “*especially happy*” or describing meeting men as a “*privilege*”. However, they also found men challenging, and stated that men are given undue praise for seeking SHC. They likened this to praising men for doing household chores or taking part in parenting their own children.

“*We feel … we are actually mostly women who work here, and we feel the women who come here* [as patients], *they are so tremendously capable* [and] *it was good that they came here. Because for so long we have been told that we must increase the percentage, we must get more guys to come to us and we feel that it becomes, oh, it’s so good that they* [men] *seek us out and it’s good that we get good statistics*.” (FG 3, Youth clinic)

In relation to the organisational discourse, men were depicted as unaware of their sexual health needs, and HCPs were considered to be doing men a favour by helping them. Participants described coaxing men to give them necessary information. They described men as being blunt, unaccustomed to the situation, and untrained in terms of talking about sexual health and sexuality. These descriptions infantilised men and portrayed them as unsocialised in the arena. Men were ascribed positive experiences of SHC, and feelings of gratitude towards SHC and HCPs. Men who had sought SHC were described using vocabulary associated with business, fishing and hunting. They were said to have “*taken the bait*”, or to have been “*lured*” or “*caught*”, and participants described “*marketing*” therapy to men because of difficulties in encouraging them to accept or “*catch onto*” the offer. This vocabulary signalled that extra efforts were needed, that SHC was not a self-evident place for men, and that men were reluctant patients.

#### Formulating a mission to change masculinity

Participants expressed a wish that their work in SHC would contribute to changing masculinity. Key words in terms of the imagery generated included “*effort towards change*”, “*mission*” and “*new*”. HCPs described wanting to change men and encourage the right kind of masculinity in patients, concepts based on HCPs’ preferences in terms of relationally situated notions of masculinity. Masculinity was constructed as a problem for which men needed help. HCPs considered that patients themselves were often unaware of this, and that changing their masculinity would solve their problems. This was explicitly described as challenging patients’ images of masculinity by making them reflect on themselves. The “*effort towards change*” was not explicit in interactions with all patients, as most patients did not seek SHC as a result of masculinity. The implicit effort towards change was described as delivering “*new perspectives*” or “*ways of thinking*”.

“*Sometimes I think about the effort for change* [to change masculinity], *what we want to achieve isn’t always so … how do you say, possible, or that it is … Maybe we can only do this much, and then its simpler to try to deliver a new reflection or way of thinking, rather than a completely new worldview*.” (FG 7, Men’s sexual health clinic)

The effort towards change was described as a kind, charitable and beneficial act. Key words used in describing the effort towards change included “*giving*”, “*opening up*”, “*ways out*”, “*non-judgemental*”, “*less constricting*” and “*more inclusive*”. The discourse on changing masculinity constructed SHC as both threatening and a facility for rescuing masculinity. Changing masculinity was described as changing society one patient at a time, and discursively positioned HCPs as agents of change. In this discourse, the social location of masculinity in SHC was constructed as a political act, and SHC was considered to be on a mission to change masculinity. Participants reflected that the effort towards change permeated SHC, and that patients might consider these views coercive.

## Discussion

This study aimed to explore how HCPs construct the gendered social location in SHC, specifically in relation to masculinity and to masculinity as a relationally situated construct. One of our main findings was that HCPs’ discourses distanced SHC from societal norms of masculinity by presenting the latter as harmful to patients and incompatible with SHC provision. SHC was positioned in opposition to societal masculinity. A key word in this positioning was “*macho*”. Macho was used discursively to link societal masculinity with gender stereotypes and gender norms, as well as power and dominion over women, transgender people and men outside the norms of masculinity. These discourses on societal masculinity are analogous to the theory of hegemonic masculinity [64], i.e. that culturally dominant aspirational ideals shape socially acceptable practices of masculinity which uphold a patriarchal gender order. In healthcare research, hegemonic masculinity has been used to explain why men take sexual risks and avoid seeking healthcare [65–67]. Similar ideals, which reward men for taking sexual risks and avoiding SHC, seem to be used by HCPs as ways of representing masculinity within a construction of SHC which is opposed to masculinity.

Whilst HCPs expressed a shared oppositional attitude to masculinity in society, they did not express an alternative shared or formalised discourse on masculinity in SHC. In other words, they lacked a professional discourse for communicating about, and with, men in SHC. Studies have reported that professional discourse is socialised and adopted during formal education [68] and through shared clinical experiences [69, 70]. Professional groups are built through shared language [71, 72]. In the case of HCPs in SHC, the group’s shared language does not seem to include a professional discourse on men and masculinity. The absence of a professional discourse could reflect a gap in knowledge about masculinity and men’s sexual health.

HCPs used discursive strategies to compensate for the absence of a professional discourse on men and masculinities. These strategies included interrelating different orders of discourse. For example, they presented private attitudes as unprofessional but aligned with opposition to societal masculinity in SHC. They renegotiated a relationally situated understanding of masculinities by citing clinical experiences. They also minimised the importance of masculinity in SHC by claiming professional gender-blindness. Previous studies have reported that HCP interdiscursively criticises, indulges and protects masculinity in primary care in order to resolve the discrepancy between valorising masculinity as a cultural ideal and experiencing it as a barrier to health [7]. In this study, interdiscursive strategies resolved contradictions between the ideal of opposing societal masculinity, perceiving masculinity as positive in personal relationships and wanting to be professional, i.e. not associating patients’ masculinity with their personal relationships. Studies suggest that denying gender [18], and discourses based on private attitudes to gender [11], risk reproducing gendered health inequalities, and our findings indicate that HCPs use both these discourses to relate to masculinity in SHC due to the lack of a professional discourse.

HCPs’ discourses on men and masculinities in SHC constructed masculinity as a violation of feminine norms, and allotted men a secondary role, or as “the other” in relation to women. This corresponds with men in reproductive healthcare feeling invisible and superfluous [32], and perceiving themselves as the second parent [73, 74]. The process of “othering” can be understood here as making a group of patients feel different. This is enacted through interactional discourses and clinical practices, as well as through structural and organisational discourses which position groups of patients as different or difficult because they deviate from “normal” or standardised clinical routines. Such patients might be labelled as difficult because “*their values, behaviors, lifestyles, or beliefs do not align with dominant views in health care*” [75]. Othering can negatively impact care and professional relationships [76]. The attitudes of HCPs have been described as crucial in SHC provision for men [32]. However, discourses which feminise SHC position men as the other, and construct women as the norm, carry with them the risk of implicitly placing the burden of, and responsibility for sexual health in society on women.

Our findings illustrate how discourses on the waiting room portray men and masculinity as visible because they do not fit in. It has been argued [77] that geographical places need to be considered as an aspect of intersectional approaches to health, and that interaction between human and non-human actors, such as technology, organisations or places, form emergent entities with effects on social structures and health [78]. The intersection between the waiting room and masculinity creates a space where expressions of masculine gender stand out and may become hypervisible, i.e. more noticeable than other bodies, in the same way that non-white bodies are hypervisible in white spaces [79]. Although the political and personal implications of this hypervisibility are completely different, the underlying principles seem to be the same, i.e. that the intersection between norm violation and coded spaces makes certain bodies appear to be out of place. In terms of understanding the social location of masculinity in SHC, it could be important to take into consideration how the waiting room, as a physical place, intersects with gender.

We identified competing discourses on men who seek SHC. Organisational discourses and institutional expectations in terms of increasing the number of men seeking SHC were incorporated into professional and private discourses, or clashed with them. Previous research suggests [80] that HCPs position themselves subjectively as either serving dominant organisational discourses, or subverting or challenging them. We found that interdiscursive practices were used to renegotiate power and create multiple subjective positions. Men were constructed as reluctant patients, and HCPs considered themselves to be making an extra effort with men. They considered themselves to have positive attitudes to men, but problematised giving men undue credit for seeking SHC in response to perceived organisational demands. In this way, HCPs could negotiate their own position in relation to the organisational discourse and institutional expectations.

In a previous study, we found that a lack of training and organisational prerequisites affected HCPs’ views on working with men’s sexual health [55]. The lack of a shared approach to men’s sexual health, and the absence of a professional discourse, indicate that training on men’s sexual health and masculinity is missing from education. This could explain why HCPs’ discourses on masculinity in SHC were formulated in relation to women and femininity as norms, and to their own private attitudes towards men and masculinity. A shared, knowledge-based discourse could enable a more consistent and shared approach to men in SHC. One reason for the lack of education on men’s sexual health could be that similar views as those expressed by the participants, i.e. that SHC was primarily regarded as feminine and aimed at women, could be held by clinical training providers, healthcare policymakers and decision-makers. The findings suggest a bias in the formal training of HCPs as neither academic nor clinical training seems to have prepared the participants for addressing men’s SHC. The results also indicate that further education on sex, gender and on sexuality and sexual health, including social and psychological aspects of sexuality and practical training on how to address potentially sensitive subjects, are needed. The knowledge presented in this study could be used by policymakers and in medical and healthcare education to address some of the structural gaps that the participants described as challenges in working with men’s SHC. Overall, the findings in this study show that, regardless of their gender, HCPs discursively construct SHC as feminine, and consider the social location of masculinity in SHC to be a potentially problematic violation of norms which HCPs aim to change. This suggests that SHC is not designed for men. The findings raise questions about how the discursive practices identified in this study could affect the ability of HCPs to provide high-quality care on equal terms, regardless of patients’ gender, and how they could influence the situation for male HCPs in SHC, particularly in relation to the othering of men as patients. More studies are needed to investigate how HCPs discourses are enacted in interaction with patients and how this affects men’s engagement with healthcare.

### Methodological considerations

The data in this study were used in a previous study on HCPs’ notions about men and masculinity [55]. Re-using qualitative data in health research has been described as “an especially fertile domain” [81], and conducting a secondary analysis of qualitative data has been recommended for investigating potentially sensitive topics [82]. Discourse analysis is a less commonly used methodology in healthcare research [56]. However, it can offer important contributions to understanding the social and political context of healthcare, and provide a breadth of knowledge on shared understanding, attitudes and perspectives among HCPs. However, further studies are needed to investigate how these discourses are enacted in interactions with patients. The analysis was an iterative process which involved returning repeatedly to the data to validate findings. To mitigate the possibility of gendered interpretations, the focus groups and analysis were conducted by researchers with different gender identities and expressions. To counteract preconceptions, and ensure reliability and transparency, the analysis was initially undertaken separately by TP and ET, and findings were discussed within the author group until consensus was reached on the naming and content of the themes.

The findings should be viewed in terms of the study’s limitations. CDA is designed to identify obstacles to social change, but cannot provide this change or offer full solutions in terms of removing the obstacles identified. The findings presented here cannot represent all HCPs working with SHC, but they provide examples of how local discourses, juxtaposed with institutional and societal discourses, establish SHC as a gendered social location. These results offer new insights into the Swedish healthcare context and should be understood in relation to the societal contexts in which they were produced, i.e., in relation to societal discourses on gender. Sweden is a country that internationally ranks comparatively high in gender equality [83, 84]. But it is also a society where gender stereotypes regarding masculinity are prevalent [85] and with a high degree of gender segregation in the work force and in educations [86]. These findings may be applicable to contexts with similar healthcare systems and gender dynamics outside Scandinavia, but studies in other societal contexts are needed to determine how transferable the results are. We assumed that discussions in groups who work together would facilitate natural conversation and self-disclosure, but as discourses on masculinity were partly relationally situated, this design could have prevented participants from speaking openly. Focus groups can be susceptible to peer consensus and social desirability. Group dynamics and power relations may influence which perspectives are presented and whose voices are heard. We tried to limit this by using probes and follow-up questions to actively encourage opposing perspectives, multiple opinions and alternative experiences. Using pre-existing groups in focus group interviews, such as work colleagues, has been linked with generating free-flowing discussions and useful data. Homogeneity among focus group participants, e.g., all participants working with men’s sexuell health at the same clinic, has also been associated with needing fewer number of focus group to gather sufficient amount of data [87]. However, it is important for moderators to be aware that there might be pre-existing power imbalances within the groups and strive to create conditions for everyone to participate on equal terms. To counteract potential power imbalances all participants were encouraged to speak freely and openly and to express any divergent or opposing opinions on the topics. Something else that is helpful in counteracting power imbalances is using two moderators [87], which we did in this study. It should be noted that the gender composition varied between focus groups. One consisted only of men, two only of women and the rest were mixed genders. It was not our intention to investigate the participants’ perceptions of, or discourses on women and femininity in SHC, nor the position of male HCPs in SHC. It is possible that an additional dimension could have been achieved in the analysis if men’s and women’s discourse practices had been analysed separately. Preferably, such study could be performed with quantitative methods and a larger study sample. During the analysis, it became clear that male participants referred to their own and their patients’ embodied experiences of SHC to a lesser degree, which might be explained by the fact that there were fewer male participants, that SHC is a feminine arena, or that it could have been difficult to represent embodied masculinity in the context. It is possible that a more balanced distribution of women and men would have resulted in other discourses than those we identified. Although participants were recruited through the SRHR network, which is coordinated by the Knowledge Centre for Sexual Health where the first author works, none of the participants or the first author had any formal role in the network or knew each other in advance. During recruitment some clinics opted to not participate citing time constraints as their reason. This was primarily PHCCs who offer drop-in times, and for whom it was difficult to allocate time for a whole team to participate simultaneously. If similar research is to be carried out in the future it is vital to find ways to ensure that HCPs working at PHCCs are given the opportunity to participate, as PHCCs are an important provider of adult and older men’s SHC, either by enabling participation outside working hours or by enabling the clinics, through planning or resources, so that more HCPs can participate. If more PHCCs had participated, it could have meant more or different perspectives.

### Conclusion

HCPs’ discourses constructed masculinity in society as incompatible with SHC, and the social location of masculinity in SHC as a violation of feminine norms. Men seeking SHC were constructed as reluctant patients, and HCPs saw themselves as agents of change with a mission to transform masculinity. The construction of gendered social locations in SHC risks othering men, in the sense that men were constructed as deviating from the normal patient. This othering runs counter to aims of providing high-quality care on equal terms, regardless of the gender of patients. HCPs lacked a shared professional discourse on masculinity in communicating with, and about men. The absence of this discourse could impact men’s access to, and experiences of SHC. A shared professional discourse could create a common foundation and a more consistent and knowledge-based approach to men in SHC. Including masculinity in the professional discourse could thus enhance conditions for men seeking SHC. As professional discourses are socialised and adopted during formal training and clinical practice, our study points to a need to include men’s sexual health and masculinity in medical education and healthcare interventions.

## Data Availability

The datasets generated during and/or analysed during the current study are available from the corresponding author, TP, upon reasonable request.

## List of Abbreviations

CDA: Critical discourse analysis
HCP: Healthcare professional
SHC: Sexual healthcare
SRHR: Sexual and reproductive health and rights

## Footnotes

1 In dialogues, participants are referred to as A, B, C, etc. in the order in which they speak. This means that person A in this quote is not the same as person A in other quotes.

2 The term is roughly similar to the term “safe space”, i.e. an area intended to be free from physical threats, bias and criticism.
